# Tafazzin Protein Expression Is Associated with Tumorigenesis and Radiation Response in Rectal Cancer: A Study of Swedish Clinical Trial on Preoperative Radiotherapy

**DOI:** 10.1371/journal.pone.0098317

**Published:** 2014-05-23

**Authors:** Surajit Pathak, Wen-Jian Meng, Hong Zhang, Sebastian Gnosa, Suman Kumar Nandy, Gunnar Adell, Birgitta Holmlund, Xiao-Feng Sun

**Affiliations:** 1 ivision of Oncology, Department of Clinical and Experimental Medicine, Faculty of Health Sciences, Linköping University, County Council of Östergötland, Linköping, Sweden; 2 epartment of Gastrointestinal Surgery, West China Hospital, Sichuan University, Chengdu, China; 3 chool of Medicine, Örebro University, Örebro, Sweden; 4 Department of Biochemistry and Biophysics, Kalyani University, Kalyani, West Bengal, India; The Chinese University of Hong Kong, Hong Kong

## Abstract

**Background:**

Tafazzin (TAZ), a transmembrane protein contributes in mitochondrial structural and functional modifications through cardiolipin remodeling. TAZ mutations are associated with several diseases, but studies on the role of TAZ protein in carcinogenesis and radiotherapy (RT) response is lacking. Therefore we investigated the TAZ expression in rectal cancer, and its correlation with RT, clinicopathological and biological variables in the patients participating in a clinical trial of preoperative RT.

**Methods:**

140 rectal cancer patients were included in this study, of which 65 received RT before surgery and the rest underwent surgery alone. TAZ expression was determined by immunohistochemistry in primary cancer, distant, adjacent normal mucosa and lymph node metastasis. *In-silico* protein-protein interaction analysis was performed to study the predictive functional interaction of TAZ with other oncoproteins.

**Results:**

TAZ showed stronger expression in primary cancer and lymph node metastasis compared to distant or adjacent normal mucosa in both non-RT and RT patients. Strong TAZ expression was significantly higher in stages I-III and non-mucinious cancer of non-RT patients. In RT patients, strong TAZ expression in biopsy was related to distant recurrence, independent of gender, age, stages and grade (p = 0.043, HR, 6.160, 95% CI, 1.063–35.704). *In silico* protein-protein interaction study demonstrated that TAZ was positively related to oncoproteins, Livin, MAC30 and FXYD-3.

**Conclusions:**

Strong expression of TAZ protein seems to be related to rectal cancer development and RT response, it can be a predictive biomarker of distant recurrence in patients with preoperative RT.

## Introduction

Tafazzin protein (TAZ) is encoded by the TAZ gene functions as a phospholipid-lysophospholipid transacylase. TAZ mutations are associated with a number of clinical disorders including Barth syndrome, dilated cardiomyopathy and endocardial fibroelastosis [1], [2]. TAZ is responsible for remodeling of cardiolipin [3] which stabilizes the assembly of respiratory chain complexes and mediates key steps in apoptosis in mitochondria. A causative link between mitochondrial dysfunction and pathological disorders has been noted in different disease models. For instance, it has been shown that mitochondrial dysfunction also plays an important role in developing of inflammation. Acehan D *et. al*. [4], pointed out that abnormal TAZ expression related mitochondrial dysfunction promotes inflammation. Wilson LD *et. al.* [5] reported that abnormal TAZ expression was related to higher IL6 expression and increased the inflammatory responses. Commonly inflammation considered as a predisposition factor for cancer, especially colorectal cancer (CRC). However, the role of TAZ expression in the oncogenic processes remains unclear. Mitochondrial dysfunction resulting in alterations of cellular signal transduction might be considered as eventual target pathways in advancing future cancer treatment. CRC is the third most common type [6] and second most cancer-related death in western world [7]. Eventhough surgery remains the curative modality for the CRC, and preoperative radiotherapy (RT) has shown a survival advantage compared to surgery alone, still the value of preoperative RT remains controversial [8], [9], [10]. These results further raised the question on whether preoperative RT has to be given more selectively or not. Now the main clinical aim is to search for predictive indicators in order to identify patients best suited for preoperative RT. Generally, potential predictive biomarkers include, for example, the expression of oncogenes and tumor suppressor genes, markers of proliferation, angiogenesis, inflammation and cell adhesion as well as regulation of genes involved in the response to RT and chemotherapy. Extensive studies are being made to improve the predictive method, including the identification of new biological and molecular marker. To identify new predictive molecules for preoperative RT and elucidate the role of TAZ protein involvement in rectal cancer patients, we investigated for the first time, the expression of TAZ protein in rectal cancer and its relationship to RT response and to clinicopathological or biological variables in the patients participating in a clinical trial of preoperative RT. Furthermore, present investigation highlights for the first time the functional interaction of TAZ with other oncoproteins by *in silico* protein-protein interaction analysis.

## Materials and Methods

### Rectal cancer patients' selection

The present study included tissue sections from 140 primary rectal cancer patients, 119 distant normal mucosa specimens (109 specimens matched to the primary rectal cancer), 79 adjacent normal mucosa specimens (70 specimens matched to the primary rectal cancer), 48 lymph node metastases (44 specimens matched to the primary rectal cancer), and 101 biopsies from the primary rectal cancer patients (91 biopsies matched to the surgical primary rectal cancer) (Figure S1).

All rectal cancer patients were from Southeast Swedish Health Care region including hospitals in Linköping, Norrköping, Jonköping, Motala, Eksjö, Varnamo and Vastervik, and they participated in the Swedish Rectal Cancer Clinical Trial of Preoperative Radiotherapy between 1987 and 1990. Initially there were 171 cancer patients. However, four patients were excluded due to surgically unresectable (advanced disease), and 27 patients had no available tissue specimen for this study. Distant normal mucosa was taken from proximal or distal margin (4–35 cm from the primary tumor) of the resected rectum, and adjacent normal mucosa was taken from the mucosa adjacent to the primary tumor, the both were histologically free from tumor. Locally curative resection was carried out in all patients.

### Experimental design

Among 140 primary rectal cancer patients, 75 patients received surgery alone, and 65 received preoperative RT followed by surgery. RT was given at a total of 25Gy in 5 fractions before surgery over a median of 6 days (5–12 days) delivered with 6–10 MV photons. Surgery was then carried out in a median of 3 days (range, 1–13 days) after RT. None of the patients received adjuvant chemotherapy before or after surgery and all patients had locally resectable rectal adenocarcinoma. Mean age of the patients at diagnosis was 67 years (range 36–86 years). There was no statistical difference between the non-RT and RT group regarding of the characteristics of the patients and tumors including gender, age, stage, differentiation, surgical type and resection margin (p>0.05; Table 1).

**Table 1:** Characteristics of the primary rectal cancer patients and tumors.

Characteristics	Non-RT (%)	RT (%)
Gender		
Male	43(57)	40(61)
Female	32(43)	25(39)
Age (year)		
≤67	31(41)	27(41)
>67	44(59)	38(59)
Stage		
I+II+III	71(94)	59(90)
IV	4(6)	6(10)
Differentiation		
Well+Moderate	62(82)	50(76)
Poor	13(18)	15(24)
Surgical type		
Anterior resection	35(47)	41(63)
Abdominoperineal	40(53)	24(37)
Resection margin		
Tumor free	71(95)	61(94)
Tumor positive	4(5)	4(6)
To anal verge (cm)		
Mean	7.7	8.4

### Ethic statement

All patients were from the South-East Swedish Health Care region and participated in a randomized Swedish rectal cancer trial of preoperative RT between 1987 and 1990. Each patient was provided with detailed information about the study aims and protocol, and gave their written informed consent prior to enrollment. Follow-up was carried out until 2004. The median follow-up period was 75 months (range, 0–193 months). The study was approved by the Institutional Review Board of the Linköping University, Sweden.

### Tissue micro-array slide preparation

All the specimens including normal mucosa, primary cancers and lymph node metastases were paraffin embedded for tissue microarray. Three morphologically representative regions were chosen in each block and 3 cylindrical core tissue specimens (0.6 mm in diameter) were taken from these areas, inserted in a recipient paraffin block.

### Immunohistochemistry

Tissue microarray sections were incubated at 60°C for overnight, deparaffinized and hydrated. The activity of endogenous peroxidase was blocked in 3% H_2_O_2_ in methanol. Nonspecific background staining sections were immersed in PBS containing 1% BSA and incubated with protein block solution (Spring Bioscience, Pleasanton, CA) for 10 minutes. The sections were then incubated with the primary rabbit polyclonal anti-TAZ antibody (HPA039557, Sigma-Aldrich Co, St. Louis, MO) at a dilution of 1∶10 in antibody diluent (DAKO, Cytomation; Glostrup, Denmark) and incubated at 4°C for overnight. After washing with PBS, a biotinylated secondary anti-rabbit secondary antibody (DAKO, lot 00082892) at a dilution of 1∶2000 was added. After 30 minutes the sections were rinsed with PBS and incubated in AB enzyme reagent (ABC Staining System, Santa Cruz Biotechnology, CA) containing avidin and biotinylated horseradish peroxidase. The slides were rinsed in PBS and incubated for 10 minutes in peroxidase substrate containing 3, 3-diaminobenzidine (DAB) chromogene and counterstained with haematoxylin. In all runs, negative controls were included.

### Immunohistochemical scoring

The immunostaining was scored by two independent observers based on the intensity of protein expression without knowledge of clinicopathological and biological information. In the case of discrepancy in individual scores, both investigators re-evaluated the slides together and reached a consensus before combining the individual scores. To avoid an artificial effect, the cells on the margins of the sections and in areas with poor morphology were not counted. The intensity of the staining was classified as negative, weak, moderate and strong staining. For statistical analyses, negative, weak or moderate stained cases were considered as low expression group, whereas strong cases were considered as high expression group. The expression of meningioma activated protein (MAC30) [11], FXYD-3 [12] and Livin [13] in rectal cancers determined by immunohistochemistry were taken from previous studies from our laboratory conducted with the same patient material used in the present study.

### *In silico* protein-protein interaction studies

Amino acid sequences, excluding signal peptide region, of TAZ, Livin (Isoform 1 & 2), FXYD-3 (Isoform 1) and MAC30 [UniProt IDs: Q16635, Q96CA5, Q14802 and Q5BJF2 respectively] and their general and sequence annotations were collected from UNIPROT.

### Template selection and model building

All amino acid sequences were subjected to NCBI PSI-BLAST (Position Specific Iteration-Basic Local Alignment Search Tool) (http://blast.ncbi.nlm.nih.gov/Blast.cgi) against PDB (Protein Data Bank) (http://www.rcsb.org/pdb/home/home.do) proteins with default parameters to select the template protein and then the templates were downloaded from PDB. Refined three-dimensional homology models were generated using a comparative modeling program MODELLER [14] of Discovery Studio (DS), based on given template-target alignment and selected template structure. Five structures were generated per protein and model with lowest value of the normalized discrete optimized protein energy was chosen as the best model. Due to unavailability of template structure with E-value better than threshold, TAZ and MAC30 was modeled by fold recognition method from I-TASSER server [15]. All the models were subjected to 2000 step energy minimization in DS Smart Minimizer in order to relieve the steric clashes. Further, the stereochemistry and conformations of the modeled structures were validated through Structural Analysis and Verification Server (SAVES) at http://nihserver.mbi.ucla.edu/SAVES/ and also with Atomic NonLocal Environment Assessment (ANOLEA) [16], ProSA [17] and MolProbity [18] servers.

### Molecular docking studies

To consider the protein-protein interaction of Livin, FXYD-3 and MAC30 with TAZ, all three proteins were docked with TAZ using rigid-body docking algorithm ZDOCK [19] in DS 2.5. Only cytoplasmic regions of FXYD-3 and MAC30 were considered for interaction studies. A total of 2000 docked poses were generated for each of the TAZ complexes which were ranked by ZDOCK score according to shape complementarity, electrostatics and desolvation energy and further, the poses were re-ranked by ZRANK [19], rescoring method employing a weighted energy function. The best 30 poses according to ZRANK score were subjected to RDOCK [19] - a CHARMm (http://www.charmm.org/) based procedure for refinement and scoring. Position optimizations of these 30 poses, were carried out with CHARMm force field, Momany-Rone [20] partial charges using DS Smart Minimizer. Relative percentage of solvent accessibility, hydrogen bond (HB) and binding free energies of TAZ complexes were also determined at physiological ionic strength using Generalized Born approximation with Molecular Volume integration [21] in DS 2.5.

### Statistical analysis

Chi-square or McNemar's test was applied to examine the significance of the differences in TAZ expression in distant or adjacent normal mucosa, primary cancer and lymph node metastasis, as well as the correlation of TAZ expression with clinicopathological or biological variables. TAZ expression with disease free survival or overall survival and distant recurrence was tested by using Kaplan-Meier analysis and Cox proportional hazards regression analysis. Survival curves were computed according to Kaplan-Meier method. All tests were two sided and p-value of <0.05 was considered statistically significant.

## Results

### TAZ showed higher expression in primary cancer and lymph node metastasis

**Figure 1:**
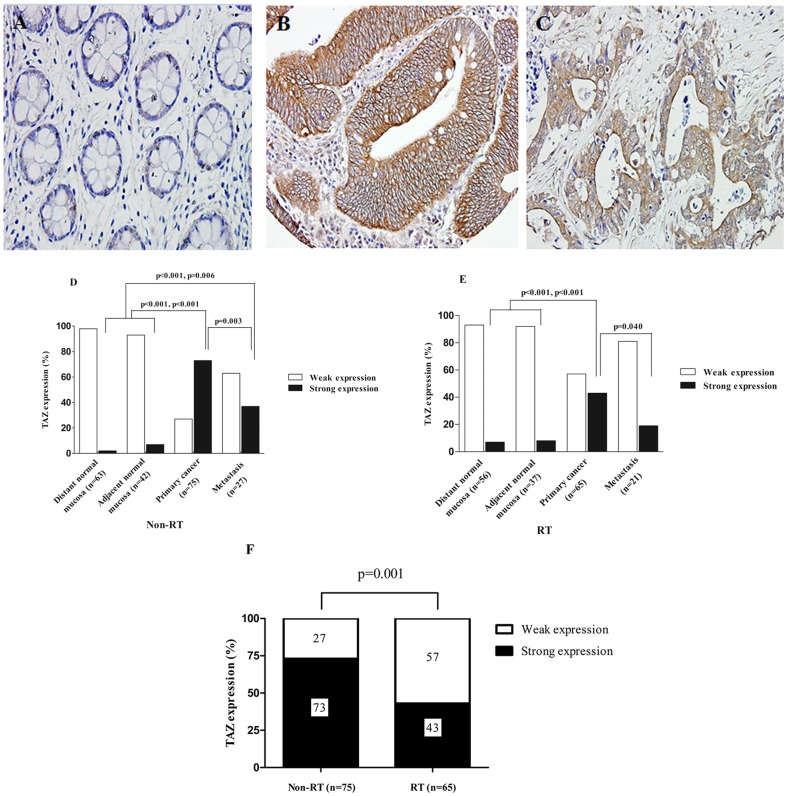
Immunohistochemical staining pictures of the TAZ protein expression and TAZ expression patterns in the non-RT and RT group. TAZ protein expression was shown in (A) normal rectal mucosa (negative expression), (B) primary cancer (strong expression) and (C) lymph node metastasis (weak expression) (A–C, 400X). In non-RT group (D) TAZ expression was significantly higher in primary cancer compared to the distant normal mucosa (p<0.001), the adjacent mucosa (p<0.001). The expression in the lymph node metastasis TAZ expression was also significantly higher compared to the expression in distant normal mucosa (p<0.001), the adjacent mucosa (p = 0.006). However, compared to primary cancer TAZ expression in lymph node metastasis was significantly lower (p = 0.003). The RT group (E) showed significantly higher TAZ expression in the primary cancer compared to the distant and adjacent normal mucosa (p<0.001 and p<0.001 respectively). In the lymph node metastasis strong TAZ expression was significantly lower compared to the expression in primary cancer (p = 0.040). Only p-values<0.05 are presented. In primary cancer strong TAZ expression was significantly lower (p = 0.001) compared to non-RT group (F).

TAZ expression was analyzed by immunohistochemistry (Figure 1, A–C) in the distant normal mucosa, adjacent normal mucosa, primary cancer and lymph node metastasis from surgical resection samples and in biopsy (corresponding to surgical primary cancer). In non-RT group (Figure 1D), TAZ showed higher expression in primary cancer (p<0.001 and p<0.001, respectively) and lymph node metastasis (p<0.001 and p = 0.006, respectively) as compared to distant and adjacent normal mucosa. As shown in Figure 1D, only 2% of the 63 distant normal mucosa samples, and 7% of the 42 adjacent normal mucosa specimens had strong TAZ expression, whereas 73% of 75 primary cancers and 37% of 27 metastases showed strong TAZ expression. In the RT group (Figure 1E), TAZ expression was increased in primary cancer (p<0.001, p<0.001) and lymph node metastasis (p = 0.130, p = 0.220) compared to distant and adjacent normal mucosa. As shown in Figure 1E, the strong TAZ expression was found in 7% of the 56 distant normal mucosa samples, in 8% of 37 adjacent normal mucosa samples, in 43% of the 65 primary cancers and in 19% of 21 metastases. However, strong TAZ expression marginally decreased from primary cancer to metastasis (p = 0.003, p = 0.040) both in non-RT and RT group. In the biopsy, the TAZ expression pattern was in line with the surgical primary cancer.

### TAZ expression decreased in primary rectal cancer after RT

Compared to non-RT group, TAZ expression in primary cancer samples subjected to RT was significantly reduced (p = 0.001). TAZ expression showed higher frequency in 73% patients in non-RT group, whereas in RT group it decreased to 43% (Figure 1F). Strong TAZ expression in metastasis also decreased in the RT group compare to non-RT group although the difference was not statistically significant (p = 0.173).

### TAZ expression in relation to clinicopathological variables in the patients

In non-RT group, TAZ expression was significantly related to stage (p = 0.031) and non-mucinous histological type (p = 0.004). It has shown that 75% of the patients with strong TAZ expression were stages I-III whereas 25% were stage IV. Furthermore 79% of the patients with strong TAZ expression were non-mucinous carcinomas while 21% were mucinous carcinoma (Table 2). In RT group, there was no such relationship (Table 2). Distant recurrence was found in 57 patients (41%) out of 140 primary rectal cancer patients.

**Table 2:** Correlation of TAZ expression with clinicopathological features of rectal cancer patients.

Clinicopathological features	Non-RT			RT		
	Weak (%)	Strong (%)	p-value	Weak (%)	Strong (%)	p-value
Gender						
Male	13(30)	30(70)	0.617	23(58)	17(42)	0.905
Female	8(25)	24(75)		14(56)	11(44)	
Age (year)						
≤67	8(26)	23(74)	0.722	16(59)	11(41)	0.748
>67	13(29)	31(61)		21(55)	17(45)	
TNM stage						
I+II+III	18(25)	53(75)	0.031	32(54)	27(46)	0.170
IV	3(75)	1(25)		5(83)	1(17)	
Differentiation						
Well+Moderate	15(24)	47(76)	0.108	29(58)	21(42)	0.748
Poor	6(46)	7(54)		8(53)	7(47)	
Mucinous						
Negative	13(21)	48(79)	0.004	29(57)	22(43)	0.854
Positive	7(64)	4(36)		6(60)	4(40)	

Varied number in the different variables is due to missing data.

### TAZ expression in relation to biological variables in the patients

In primary tumors, TAZ expression was positively correlated with MAC30 expression (p = 0.022) in the RT group (Table 3). Furthermore, in both non-RT and RT group strong TAZ expression was positively correlated with FXYD-3 (p = 0.003 and p = 0.008, respectively), as well as with the Livin expression (p = 0.041 and p = 0.013 respectively, Table 3).

**Table 3:** Correlation of TAZ expression with biological factors in rectal cancer patients with and without radiotherapy (RT).

Biological factors	Non-RT			RT		
	Weak (%)	Strong (%)	p-value	Weak (%)	Strong (%)	p-value
MAC30						
Weak	6(23)	20(77)	0.418	13(76)	4(24)	0.022
Strong	12(32)	25(68)		15(43)	20(57)	
FXYD-3						
Weak	14(50)	14(50)	0.003	15(79)	4(21)	0.008
Strong	6(16)	32(84)		15(42)	21(58)	
Livin						
Weak	19(35)	35(65)	0.041	36(63)	21(37)	0.013
Strong	2(11)	17(89)		1(14)	6(86)	

### Predictive significance of TAZ expression in rectal cancer for preoperative RT

**Figure 2:**
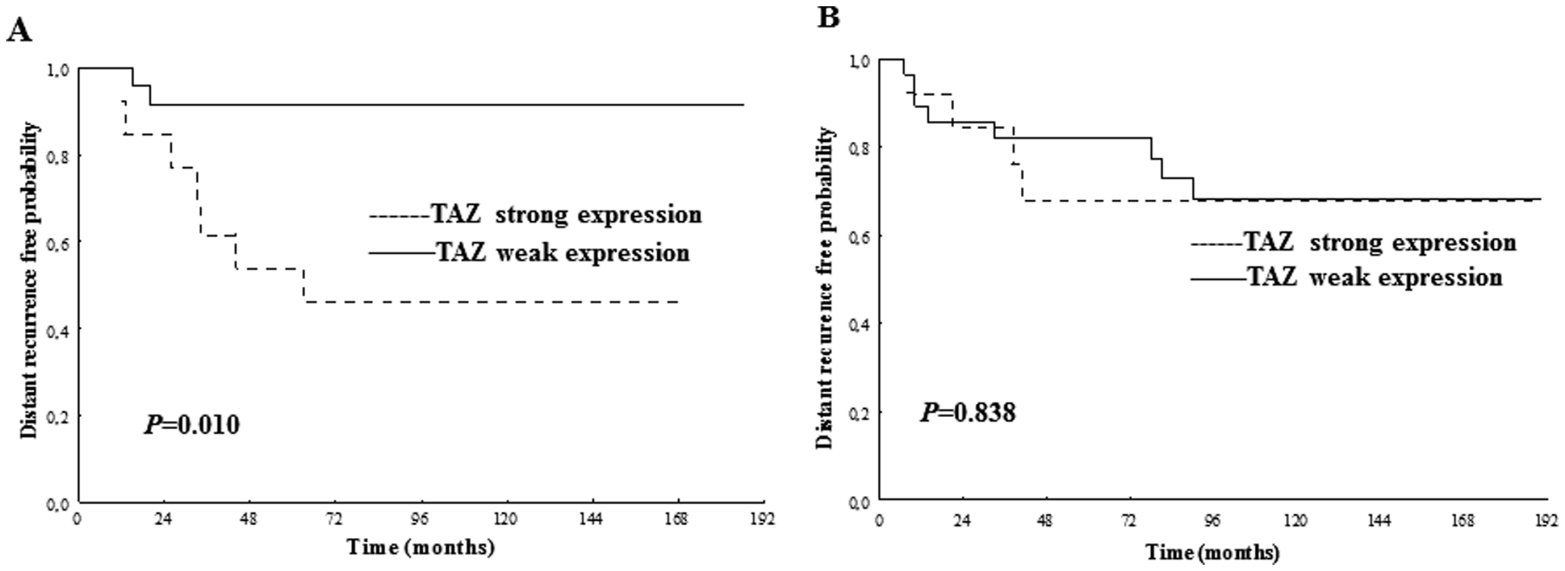
TAZ expression in relation to distant recurrence in biopsy samples. In the RT group, patients with strong TAZ expression had a significantly higher risk of developing distant recurrence (A) (p = 0.010). In the non-RT group, the TAZ expression was not significantly related to distant recurrence probability (B). Only p-values<0.05 are presented.

In biopsies, strong TAZ expression was related to distant recurrence in the RT group, *i.e.* strong TAZ expression increased the probability of distant recurrence (p = 0.010, Figure 2A). Further multivariate analysis confirmed the correlation was independent of gender, age, stage and differentiation (Table 4). In the non-RT group, no such relationship was found (Figure 2B). However, TAZ expression was not significantly related to disease-free survival and overall survival in the patients either in RT or non-RT group (Figure S2).

**Table 4:** Expression of TAZ in relation to distant recurrence probability in rectal cancer patients with preoperative RT.

Characteristics	Univariate	Multivariate	
	p-value	HR (95% CI)	p-value
TAZ expression			
Weak *vs.* Strong	0.012	6.160 (1.063–35.7036)	0.043
Gender			
Male *vs*. Female	0.157	0.228 (0.022–2.326)	0.212
Age (year)			
≤67 *vs*.>67	0.602	0.945 (0.195–4.584)	0.944
Stage			
I *vs*. II+IIIA+IIIB	0.010	8.6238 (0.891–83.5035)	0.063
Differentiation			
Well+Moderate *vs*. Poor	0.733	1.561 (0.284–8.582)	0.609

HR, hazard ratio; 95%CI, 95% confidence interval.

### *In silico* protein-protein interaction studies

#### Template selection

Except FXYD-3, PSI-BLAST failed to recognize any template with 100% query coverage both individually or in combination; and was homology modeled by using solution NMR structure PDB ID 2JO1 [Model 1, Chain A, 37.7% identity, covering sequence length 22-87]. On the other hand, the functional domains of Livin, that belong to BIR and RING superfamily, were well represented by crystallographic structures PDB ID 3F7G [Chain B, Resolution 2.30 Å, covering sequence length 71–169 of isoform 2] and PDB ID 4AUQ [Chain B, Resolution 2.18 Å, covering sequence length 242–298 of isoform 1]. The best model from I-TASSER server was taken for TAZ (C-score = −0.28, TM-score = 0.68±0.12) and MAC30 (C-score = −2.93, TM-score = 0.38±0.13).

#### Molecular docking studies

**Figure 3:**
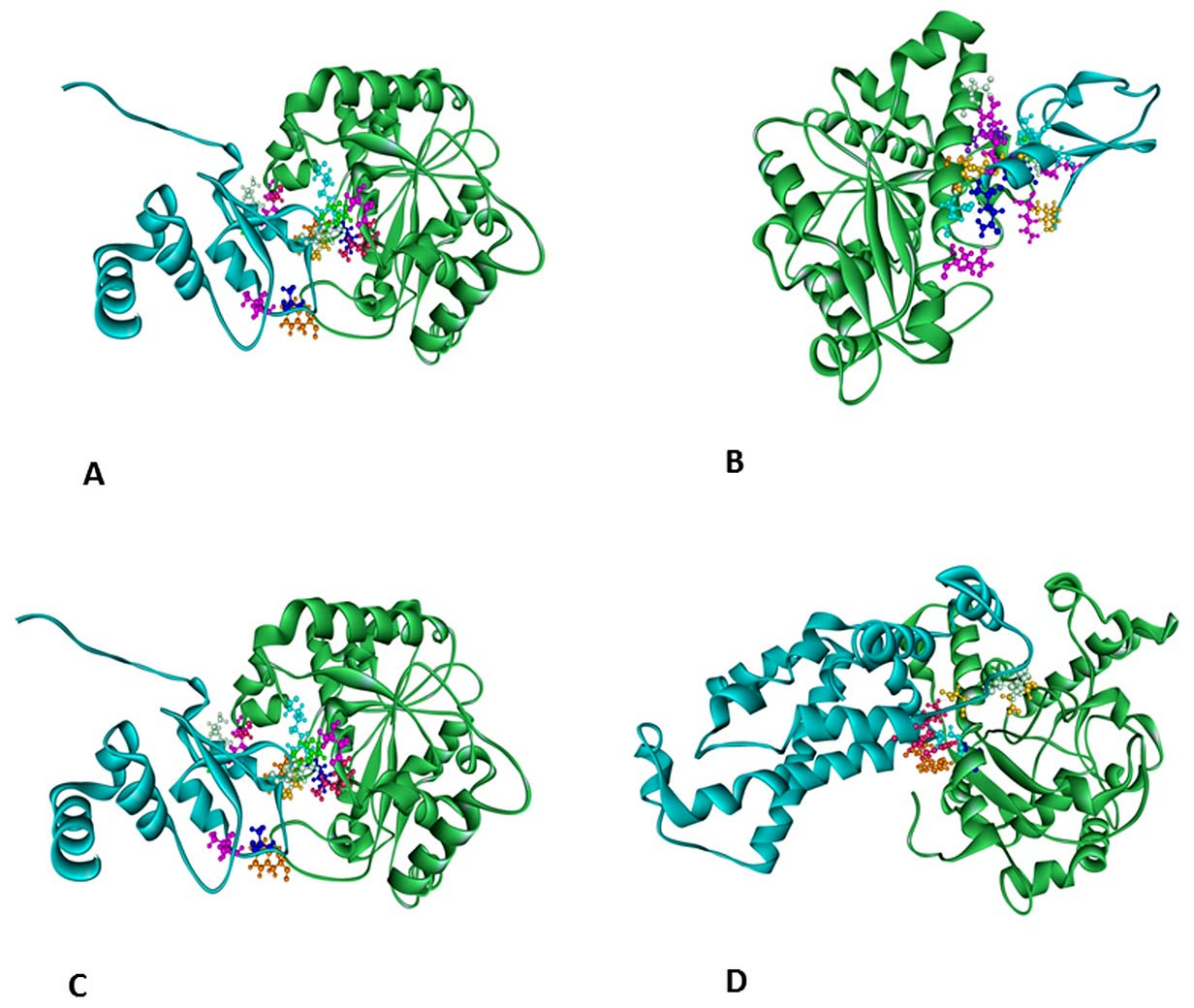
Possible conformations of the TAZ complexes. TAZ is represented in green, other proteins in cyan. A: TAZ-Livin (TAZ-BIR) complex; B: TAZ-Livin (TAZ-RING) complex; C: TAZ-FXYD-3 complex; D: TAZ-MAC30 complex.

Physical properties of protein–protein interface of all the docked complexes (Figure 3A–D) were analyzed and listed in the Table 5. The complexes were mainly characterized by polar interfaces, which indicate towards the possible non-obligatory nature of the complexes. Adequate binding surface area, recognizable free energy and their corresponding stabilization by non-bonded interactions, say hydrogen bonds and hydrophobic interactions indicate towards possible interaction between TAZ and the proteins of our interest.

**Table 5:** Interface parameters for the docked complexes.

Complex	Domains involved	Binding surface area (Å^2^)	Number of HB	% of Residues		Binding Free Energy (kcal/mol)
				Polar	Non-Polar	
TAZ-Livin	TAZ-BIR	1806.9	10	67.7	32.3	−77.5212
	TAZ-RING	1699.98	16	58.2	41.8	−53.8787
TAZ- FXYD-3	TAZ-FXYD	1364.94	4	73.8	26.2	−50.8220
TAZ-MAC30	TAZ-MAC30	1509.1	7	64.3	35.7	−46.5495

## Discussion

TAZ protein is involved in maintaining levels of cardiolipin which is essential for energy production in the mitochondria. Mutations in TAZ gene change the structure of TAZ protein and prevent it from performing its normal role in the mitochondria. It was first proposed by Otto Heinrich Warburg [22] that cancer originated from irreversible injury to mitochondrial respiration, but the structural basis for this injury has remained elusive. Recently, a study [23] shows major abnormalities in cardiolipin content in all tumors and overexpression of TAZ gene in thyroid carcinoma [24]. However, the abnormality in TAZ expression in cancer has not been investigated thoroughly till date. In the present study, we have demonstrated for the first time that TAZ expression in primary cancer increased compared to distant or adjacent normal mucosa, and then decreased in lymph node metastasis. We also found that in the non-RT group, TAZ expression was significantly higher in stages I-III tumors compared to stage IV tumors, and in non-mucinous tumors compared to mucinous tumors. Since stage IV is considered as distant metastatic tumor, and mucinous cancer is more prone to develop metastasis and aggressiveness, thereby suggesting that TAZ expression may play an important role in the development of different stage and histological types of rectal cancer.

Furthermore, strong TAZ expression was positively correlated with FXYD-3 and Livin expression in the non-RT group. It is suggested that FXYD-3 is upregulated early in the process of malignant transformation [25]. Likewise, upregulated expression of Livin has been implicated in tumorigenesis and metastasis in epithelial tumors such as colorectal cancer [26]. Since significant correlations between the TAZ expression with Livin, MAC30 and FXYD-3 were found in the same patient cohort, we did *in silico* protein-protein interactions analysis between TAZ-livin, TAZ-MAC30 and TAZ-FXYD-3. Molecular docking studies, viz. predicted binding free energy, binding surface area etc., of the best poses as well as their average values for all top thirty poses of each complex also points towards possible functional TAZ interaction with the mentioned oncoproteins. Furthermore, Makaryan *et. al*., [27] observed increase in apoptotic response after TAZ knock-down. So TAZ overexpression possibly is related to anti-apoptosis and abnormal cell growth. During the cell cycle, the genes for glycolytic enzymes are overexpressed at the time of active DNA synthesis [28]. Likewise, several oncogenes (c-myc, HIF1α, Akt) or tumour suppressors (p53) are involved in the transactivation of glycolytic enzymes [29]. The activation of these genes in correlation with TAZ overexpression may also involve mitochondrial dysregulation. Hence, our findings indicate that strong TAZ expression in association with other oncoproteins might be directly related to the process of rectal cancer development. Further studies to elucidate the link between TAZ and these oncoproteins would improve our understanding of TAZ function in the rectal carcinogenesis.

Although radiotherapy together with surgery have improved the survival, there is still a large proportion of patients receiving RT without any benefits, but experiencing side effects. Advances in molecular biology have provided an opportunity to select patients suited for therapy and determine prognosis. However, so far, no single method has come to clinical use in this context, *i.e*., there is still a large gap between the laboratory discoveries and clinical practice. Interestedly, we found that strong TAZ expression in biopsies of preoperative RT group was significantly correlated with distant recurrence, independent of gender, age, tumor stages and grade. Therefore, the present investigation suggests that, TAZ, may be considered as a factor for selecting groups of rectal cancer patients for preoperative RT.

Since TAZ expression was related to tumour progression, we further investigated whether TAZ was related to the response to RT. Interestingly, we found that RT significantly lowered TAZ expression in primary rectal cancer. In lymph node metastasis, the effect of RT was not significant. These results suggest that the effect of RT decreases TAZ expression of rectal cancers was only confined to the patients with non-metastatic locally advanced rectal cancers but not metastatic cancers. Our results pointed that strong TAZ expression in the patients those underwent preoperative RT was positively correlated with FXYD-3, Livin and MAC30 expression, and further TAZ functionally interacted with these proteins thereby indicating that TAZ may act together with FXYD-3, Livin and MAC30 in response of the radiation.

In conclusion, the strong expression of TAZ was involved in the development of rectal cancer and radiation seemed to decrease the TAZ expression of primary rectal cancers. Moreover, TAZ was an independent predictive marker of distant recurrence in rectal cancer patients with preoperative RT, Hence, selection of patients for preoperative RT and also targeting TAZ protein by using TAZ antagonist along with preoperative RT may be a better treatment option for rectal cancer.

## Supporting Information

Figure S1 Profiles of the rectal cancer patients including biopsies (taken before surgery) and surgical samples of distant normal (DN), adjacent normal (AN), primary tumor (PT) and lymph node metastatic tumor (MT).
(TIF)

Figure S2 Relationship between TAZ expression and overall (A, C) or disease free survival (B, D) in rectal cancers patients. A and C represents the RT group, B and D the non-RT group.
(TIF)
