# Supplementary figures and images for: Tafazzin Protein Expression Is Associated with Tumorigenesis and Radiation Response in Rectal Cancer: A Study of Swedish Clinical Trial on Preoperative Radiotherapy
Source: PLoS One. 2014 May 23;9(5):e98317. doi: 10.1371/journal.pone.0098317 (PMC4032294; doi:10.1371/journal.pone.0098317)

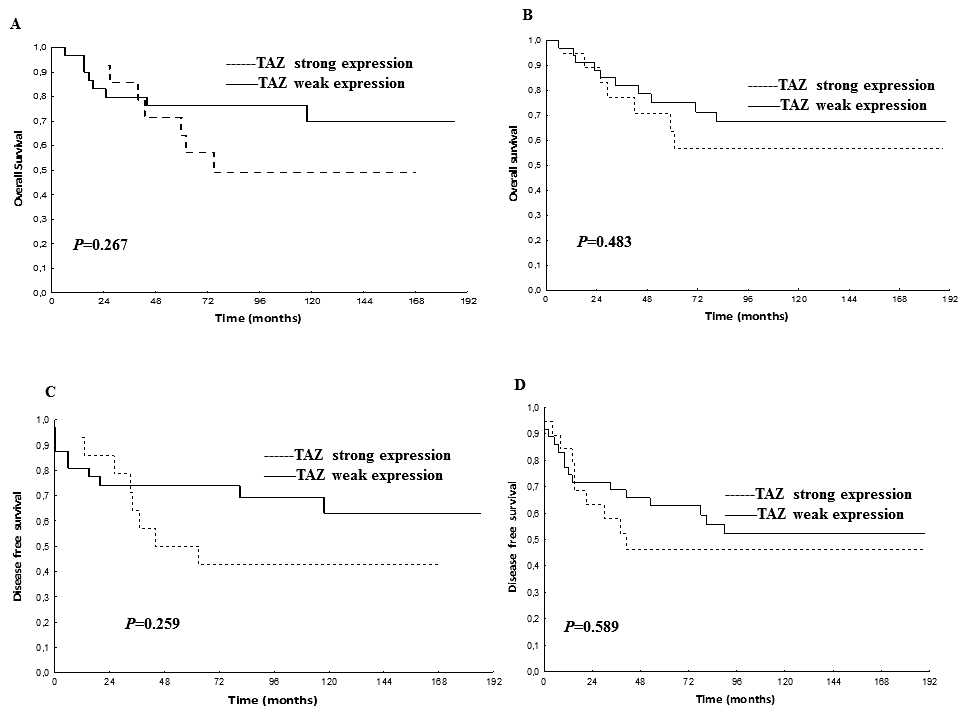

Supplement: Figure S2 — Relationship between TAZ expression and overall (A, C) or disease free survival (B, D) in rectal cancers patients. A and C represents the RT group, B and D the non-RT group. (TIF) [file pone.0098317.s002.tif]
